# Defining the extreme substrate specificity of *Euonymus alatus* diacylglycerol acetyltransferase, an unusual membrane-bound *O*-acyltransferase

**DOI:** 10.1042/BSR20160277

**Published:** 2016-11-08

**Authors:** Sunil Bansal, Timothy P. Durrett

**Affiliations:** * Department of Biochemistry and Molecular Biophysics, Kansas State University, 141 Chalmers Hall, Manhattan, KS 66506, U.S.A.

**Keywords:** acetyl-TAGs, acetyltransferase, MBOAT, substrate specificity, triacylglycerols

## Abstract

*Euonymus alatus* diacylglycerol acetyltransferase (*Ea*DAcT) synthesizes the unusually structured 3-acetyl-1,2-diacylglycerols (acetyl-TAG) found in the seeds of a few plant species. A member of the membrane-bound *O*-acyltransferase (MBOAT) family, *Ea*DAcT transfers the acetyl group from acetyl-CoA to *sn*-1,2-diacylglycerol (DAG) to produce acetyl-TAG. *In vitro* assays demonstrated that the enzyme is also able to utilize butyryl-CoA and hexanoyl-CoA as acyl donors, though with much less efficiency compared with acetyl-CoA. Acyl-CoAs longer than eight carbons were not used by *Ea*DAcT. This extreme substrate specificity of *Ea*DAcT distinguishes it from all other MBOATs which typically catalyze the transfer of much longer acyl groups. *In vitro* selectivity experiments revealed that *Ea*DAcT preferentially acetylated DAG molecules containing more double bonds over those with less. However, the enzyme was also able to acetylate saturated DAG containing medium chain fatty acids, albeit with less efficiency. Interestingly, *Ea*DAcT could only acetylate the free hydroxyl group of *sn*-1,2-DAG but not the available hydroxyl groups in *sn*-1,3-DAG or in monoacylglycerols (MAG). Consistent with its similarity to the jojoba wax synthase, *Ea*DAcT could acetylate fatty alcohols *in vitro* to produce alkyl acetates. Likewise, when coexpressed in yeast with a fatty acyl-CoA reductase capable of producing fatty alcohols, *Ea*DAcT synthesized alkyl acetates although the efficiency of production was low. This improved understanding of *Ea*DAcT specificity confirms that the enzyme preferentially utilizes acetyl-CoA to acetylate *sn*-1,2-DAGs and will be helpful in engineering the production of acetyl-TAG with improved functionality in transgenic plants.

## Abstract (precis)

The membrane-bound O-acyltransferase *Euonymus alatus* diacylglycerol acetyltransferase (*Ea*DAcT) preferentially uses acetyl-CoA to acetylate *sn*-1,2 DAGs but other acyl-donor and acyl-acceptor substrates can be used with low efficiency.

## INTRODUCTION

The enzyme *Euonymus alatus* diacylglycerol acetyltransferase (*Ea*DAcT) synthesizes 3-acetyl-1,2-diacylglycerols (acetyl-TAG) by transferring an acetyl group from acetyl-CoA to the *sn*-3 position of diacylglycerol (DAG) [1]. Acetyl-TAG are unusually structured triacylglycerols (TAG) found in the seeds of various plant species, including *E. alatus* [2–4] and possess different properties than typical TAG due to the presence of the *sn*-3 acetate group. For example, acetyl-TAG possess a lower kinematic viscosity and improved cold temperature properties, making them potentially useful for a variety of applications [1,5].

*Ea*DAcT belongs to the membrane-bound *O*-acyltransferase (MBOAT) family of enzymes, which catalyze the acyl-CoA dependent acylation of hydroxyl groups present on lipids such as cholesterol and DAG [6,7]. For example, type 1 diacylglycerol acyltransferases (DGAT1) use long-chain acyl-CoAs to acylate DAG to form TAG [8]. Other family members are capable of acylating amino acid residues on small peptides and larger protein substrates. The acyltransferase Porcupine esterifies palmitoleic acid to a serine residue on murine Wnt-3a to enable the extracellular secretion of this member of the Wnt family of secreted signalling proteins important for cell-to-cell interaction [9]. *Ea*DAcT's acyl donor specificity is therefore unusual compared with other MBOATs in that it uses the shortest possible acyl-CoA and not longer acyl-CoAs [1]. Likewise, *Ea*DAcT's ability to acylate DAG is somewhat surprising as its protein sequence is more similar to enzymes that acylate fatty alcohols and sterols than to DGAT1 [1,10]. Consistent with this similarity to the jojoba wax synthase, we previously demonstrated that *Ea*DAcT can weakly acetylate fatty alcohols to produce alkyl acetates. Here *Ea*DAcT was shown to be able to synthesize a range of medium chain alkyl acetates, which can act as insect pheromone-like compounds [11,12].

However, beyond the work investigating the ability of *Ea*DAcT to acetylate different fatty alcohols, much remains unknown about the substrate specificity of the enzyme. Although initial *in vitro* studies demonstrated the enzyme uses acetyl-CoA but not oleoyl-CoA, *Ea*DAcT's ability to use acyl-CoAs with acyl groups containing 2–18 carbons was not determined. Likewise, little is known about the enzyme's preference for different DAG molecules and other potential glycerolipid acyl acceptors. Here we extend what is known about the substrate specificity of *Ea*DAcT, both in terms of its acyl-donor and acyl-acceptor substrate preferences.

## EXPERIMENTAL

### Lipid reagents and standards

Unlabelled acyl-CoAs and [1,2-^13^C] acetyl-CoA were obtained from Sigma–Aldrich. [1-^14^C] acetyl-CoA was purchased from PerkinElmer Life Sciences. Fatty alcohol, TAGs and alkyl acetates were obtained from Nu-Check Prep. The synthesis of acetyl-TAG, butyryl-TAG and hexanoyl-TAG standards for ESI-MS-based quantification has been described previously [13]. Different detergents were obtained from a Surfact-Amps Detergent Sampler (Fisher Scientific).

### Optimization of *Ea*DAcT activity assays

Microsomes from INVSc1 yeast expressing *Ea*DAcT were isolated, and temperature and pH optimization assays for *Ea*DAcT activity using [1-^14^C] acetyl-CoA were conducted, as previously described [1] except that no exogenous DAG was added. As INVSc1 contains endogenous DAG acyltransferases that could interfere with substrate specificity experiments, further assays were performed with microsomes from the *Saccharomyces cerevisiae* quadruple knockout strain H1246 [14] expressing *Ea*DAcT. Assays for the optimization of microsomal protein were incubated for 30 min. Incubation time optimization assays contained 80 μg microsomal protein. Both sets of experiments were incubated at 30 °C at a pH of 7.5.

### Acyl-CoA specificity assays

Microsomes isolated from H1246 yeast expressing *Ea*DAcT were incubated without any added acyl-CoA or with 250 μM unlabelled acetyl-CoA, butyryl-CoA, hexanoyl-CoA, octanoyl-CoA, decanoyl-CoA, dodecanoyl-CoA, tetradecanoyl-CoA, hexadecanoyl-CoA or octadecenoyl-CoA. After quenching the assay, 150 pmol 3-acyl-1,2-dipentadecanoin internal standard with the same *sn*-3 acyl group as the acyl-CoA [13] was added before lipid extraction to achieve a final concentration of 250 nM when the extract was dissolved in 600 μl of chloroform. TAGs formed during the reaction were detected using an ESI-MS neutral loss scan for the appropriate *sn*-3 acyl group and spectral peak areas were deconvoluted and corrected for isotopic overlap and variation as described previously [13]. Processed signals obtained for different TAG molecular species were normalized to the 3-acyl-1,2-dipentadecanoin internal standard. Values obtained from samples without added acyl-CoA were subtracted as background. For detailed kinetic studies, *Ea*DAcT microsomes were incubated without acyl-CoA or with [1,2-^13^C] acetyl-CoA, butyryl-CoA or hexanoyl-CoA at concentrations varying from 50 to 1500 μM with the same batch of microsomes. Sixty pmol 3-acyl-1,2-dinonadecanoin was added as an extraction standard prior to lipid extraction and 75 pmol 3-acyl-1,2-dipentadecanoin was added as a technical standard after extraction to achieve final concentrations of 100 and 125 nM, respectively, in 600 μl of chloroform. Reaction products for all three acyl-CoAs were quantified using ESI-MS as described previously [13].

### DAG specificity assays

Total lipid extracts from yeast microsomes expressing *Ea*DAcT were separated on Silica gel 60 TLC plates impregnated with boric acid, using a chloroform:acetone 80:10 (v/v) solvent system and visualized by staining with 0.075% (w/v) 2,7-dichlorofluorescein in 95% (v/v) methanol and viewing under UV light (312 nm). Endogenous *sn*-1,2-DAG was recovered by scraping the silica and eluting the lipids with 1:1 (v/v) hexane:diethyl ether, which were then chemically acetylated using acetic anhydride and pyridine [15]. The resulting acetyl-TAG was quantified as described previously [13].

### Alcohol acetyltransferase assays

Prior to the assay, fatty alcohols were added to the bottom of the reaction tubes and dissolved in 15 μl DMSO. Microsomes were added and the tube was incubated on ice for 5 min to allow the microsomes to absorb the fatty alcohols. Eighty microlitres reaction buffer [1] was added and the reaction was initiated by adding 5 μl [1-^14^C] acetyl-CoA (final concentration=125 μM). Reactions were quenched using hot propan-2-ol and lipids were extracted and separated using TLC as described previously [1]. Oleyl acetate was used as a reference standard. Alkyl acetate products were quantified by scraping the appropriate band and using liquid scintillation counting.

### Heterologous expression in *Saccharomyces cerevisiae*

The expression of *Ea*DAcT in the *S. cerevisiae* quadruple knockout strain H1246 [14] has been described previously [1]. The plasmid pPT534 containing *Apis mellifera* fatty acyl-CoA reductase 1 (*Am*FAR1) in the yeast expression vector pESC-URA [16] was kindly provided by Dr Xiao Qiu (University of Saskatchewan). To coexpress *Ea*DAcT, the open reading frame with a C-terminal haemagglutinin epitope was amplified using the primers 5′-AT-GCGGCCGCGATGGATGCTCATCAAGAGATCAAG-3′ and 5′-GGAAGATCTCACAAATCCCATGTAGGA-3′ and then digested with NotI and BglII. The amplified fragments were cloned into corresponding sites of the yeast expression vectors pESC-URA and pPT534 to obtain the pESC-URA-*Ea*DAcT-HA single expression and pESC-URA-*Am*FAR1-*Ea*DAcT-HA coexpression vectors respectively. The yeast strain H1246 was transformed with empty vector pESC-URA, pESC-URA-*Am*FAR1 (pPT534), pESC-URA-*Ea*DAcT-HA and pESC-URA-*Am*FAR1-*Ea*DAcT-HA. Transformed cells were grown in synthetic medium lacking uracil and tryptophan with 2% (w/v) galactose at 30 °C for 72 h to obtain a *D*_600_ of 3.2–3.4. The yeast cells were collected from 50 ml culture and lyophilized to determine their dry weight. Lipids were extracted using an established chloroform–methanol extraction method [1] and resuspended in 1.0 ml of toluene.

### Lipid analysis

To quantify alkyl acetates, yeast lipid extracts were separated on Silica gel 60 thin layer chromatography (TLC) plates (Merck) with a hexane:diethyl ether:acetic acid, 70:30:1 (v/v/v) solvent system and visualized by spraying with 0.01% (w/v) primuline in 80% (v/v) acetone and observing under UV light (312 nm). 8.5 nmol pentadecyl acetate was added to the alkyl acetate bands that comigrated with an oleyl acetate reference standard. Alkyl acetates were recovered by scraping and extracting the silica with hexane. Fatty alcohols were quantified by first transmethylating total lipid extracts using an acid catalysed method [17] to convert glycerolipids to fatty acid methyl esters (FAMEs). Fatty alcohols were then derivitized in 100 μl *N*,*O*-Bis(trimethylsilyl)trifluoroacetamide and 100 μl pyridine at 110 °C for 10 min. Heptadecanol was used as an internal standard. FAMEs, fatty alcohols and alkyl acetates were quantified using an Agilent Technologies 6890N gas chromatograph equipped with a DB-5ms (0.25 mm × 60 m) column and an Agilent Technologies 5975 inert XL mass selective detector. The injector was operated in splitless mode with an injection volume of 2.0 μl. The carrier gas was helium with a total flow rate of 9.0 ml·min^−1^. The oven temperature was maintained at 60 °C for 2 min and then ramped to 200 °C at 50 °C·min^−1^, kept there for 1 min, then ramped to 280 °C at 10 °C·min^−1^ and kept there for 3 min. The mass quad was maintained at 150 °C with a source temperature of 230 °C. The detector was set to detect the total ion count of all fragment ions with a mass range between 50 and 650. FAMEs, fatty alcohols and alkyl acetates were identified by matching their fragmentation patterns against reported spectra present in the NIST database.

## RESULTS AND DISCUSSION

### Optimization of assay conditions

To maximize product accumulation, we optimized the conditions for performing diacylglycerol acetyltransferase assays with microsomal *Ea*DAcT. Varying the incubation temperature and the pH of the reaction buffer revealed maximum acetyl-TAG accumulation at 30 °C (Supplementary Figure S1A) and a pH optimum of 7.5 (Supplementary Figure S1B); these conditions were used for all subsequent assays. Product accumulation was linear with increasing total microsomal protein up to 100 μg (Supplementary Figure S1C). Hence, to keep the reaction in a linear range for kinetic assays but to still achieve reasonable product levels, 80 μg microsomal protein was used for further assays. With this amount of microsomal protein, the acetyl-TAG product formation increased sharply during the first 5 min of the reaction after which the accumulation slowed and was essentially unchanged after 20 min (Supplementary Figure S1D). Therefore, to maximize product accumulation particularly for substrates utilized less efficiently, an incubation time of 25 min was selected for further work.

### *Ea*DAcT is capable of using other short-chain acyl-CoAs as acyl donors

**Figure 1:**
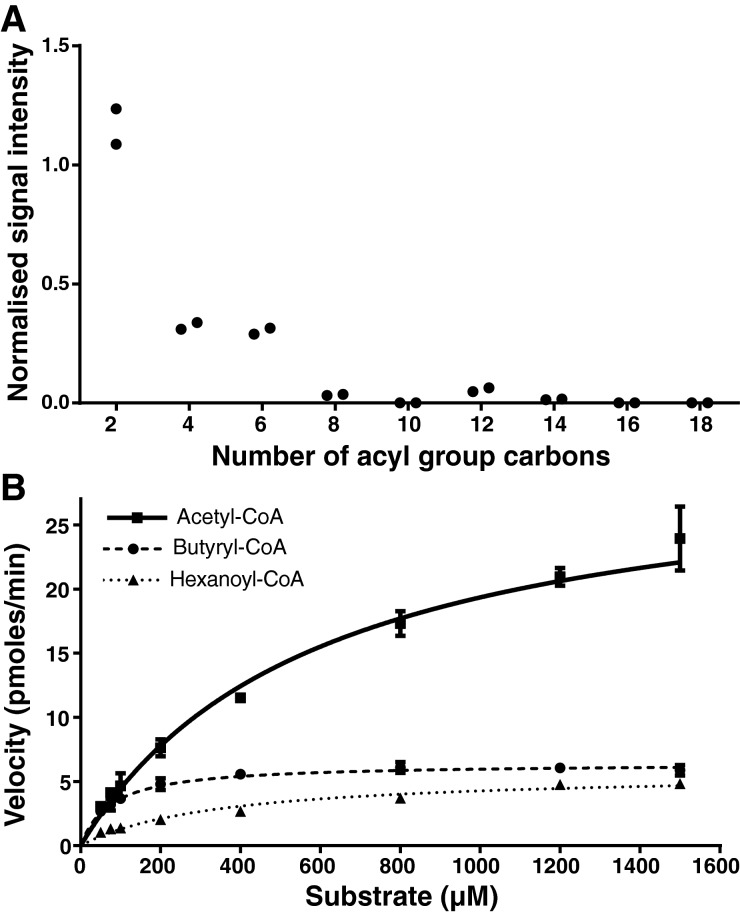
*Ea*DAcT utilizes very short-chain acyl-CoA molecules (**A**) Yeast microsomes expressing *Ea*DAcT were incubated with 250 μM of acyl-CoA with different acyl chain lengths. Reaction products were detected using ESI-MS scans for the neutral loss of the *sn*-3 acyl group and normalized to a 3-acyl-1,2-dipentadecanoyl-*sn*-glycerol standard that possessed the same *sn*-3 acyl group. Duplicate reactions were performed for each acyl-CoA substrate. These results are representative of at least three independent experiments performed on microsomes isolated at different times. (**B**) The acyltransferase activity of *Ea*DAcT using different short chain acyl-CoA substrates was determined across a range of substrate concentrations. Reaction products were quantified using ESI-MS scans for the neutral loss of the *sn*-3 acyl group. Values are expressed as mean ± S.D. for three independent assays. Curves were fitted using non-linear regression (GraphPad Prism).

Previously we showed that *Ea*DAcT uses acetyl-CoA but not oleoyl-CoA as its acyl donor substrate [1]. Given this extreme substrate specificity, we were interested to determine whether the enzyme could use acyl-CoAs intermediate in length between acetyl-CoA and oleoyl-CoA. We initially screened the acyl-CoA preference of *Ea*DAcT by incubating microsomes expressing the enzyme with different acyl-CoAs varying in carbon chain length from two to eighteen carbon atoms. Reaction products were detected using ESI-MS to scan for the neutral loss of the added acyl group and normalized to an internal standard that possessed the same *sn*-3 acyl group. Here, only acyl-CoA with acyl chain lengths up to six carbons showed detectable product levels (Figure 1A). Lacking a complete set of structured TAG standards with appropriate *sn*-3 acyl groups, we did not correct for ionization efficiency and thus quantitative comparisons between the accumulation of products with different *sn*-3 groups need to be made with caution. However, the data still suggest that considerably more acetyl-TAGs were produced than TAGs with *sn*-3 butyrate or hexanoate groups. Further, *Ea*DAcT showed negligible activity for acyl-CoA with acyl chains longer than eight carbon atoms, consistent with previous results where no *Ea*DAcT activity was observed for oleoyl-CoA [1]. These results distinguish *Ea*DAcT from other MBOATs which use longer acyl-CoA substrates. For example, plant DGAT1 enzymes prefer longer chain acyl-CoAs [8] though it should be noted that little work has been done to characterize the ability of this group of enzymes to use very short acyl-CoA substrates. The only other atypical MBOAT in this regard is ghrelin *O*-acyltransferase (GOAT) which octanylates the appetite stimulating peptide ghrelin to activate the hormone [18,19]. However, even compared with GOAT, the acyl donor specificity of *Ea*DAcT is more extreme with it only being able to use substrates shorter than octanoyl-CoA.

To further validate these results, the activity of *Ea*DAcT when using acetyl-CoA, butyryl-CoA or hexanoyl-CoA was quantified over a range of substrate concentrations. For these experiments, the appropriate structured TAG standards were synthesized, allowing absolute quantification of the different *Ea*DAcT reaction products [13]. Importantly, at all substrate concentrations, higher rates of production formation were always achieved with acetyl-CoA compared with butyryl- or hexanoyl-CoA (Figure 1B), strongly suggesting acetyl-CoA is the preferred substrate of the enzyme. Although these experiments were not designed to determine *K*_m_ and *V*_max_, the highest velocity observed for acetyl-CoA (23.9 pmol·min^−1^) was considerably higher than that for the other two substrates. Reaction rates with butyryl-CoA and hexanoyl-CoA were relatively similar to one another (5.9 pmol·min^−1^ for butyryl-CoA, 4.8 pmol·min^−1^ for hexanoyl-CoA), reflective of the acyl-CoA screening results (Figure 1A) and further demonstrating that *Ea*DAcT preferentially uses acetyl-CoA over even slightly longer chain length acyl-CoAs to esterify an acetyl group on to the *sn*-3 position of DAG.

### *Ea*DAcT preferentially acetylates unsaturated DAG *in vitro*

**Figure 2:**
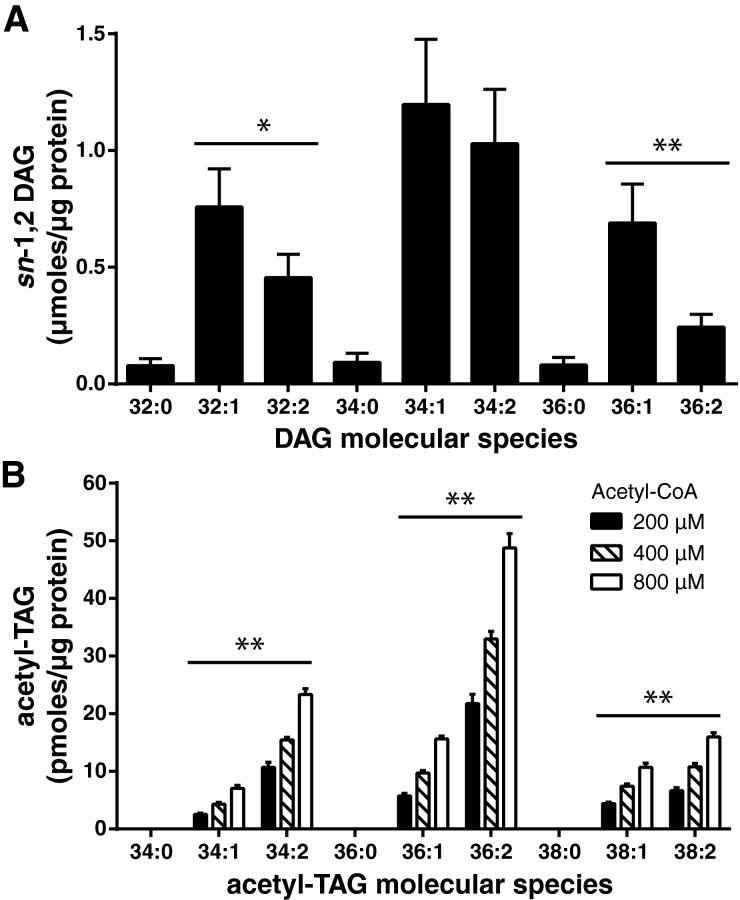
*Ea*DAcT preferentially acetylates more unsaturated DAG molecular species *in vitro* (**A**) Quantification of DAG molecular species initially present in yeast microsomes expressing *Ea*DAcT. Values represent the mean ± S.D. for three independent lipid extractions. (**B**) Quantification of acetyl-TAG molecular species formed after incubation of the same *Ea*DAcT microsomes with different concentrations of acetyl-CoA. Values represent the mean ± S.D. for three independent assays. For both DAG (**A**) and acetyl-TAG (**B**) molecular species, the number of acyl carbons (*x*) and double bonds (*y*) is indicated by *x*:*y*. Asterisks indicate statistical differences between the DAG or acetyl-TAG molecular species containing the same number of acyl carbons but different numbers of double bonds (unpaired *t*-test; *, *P*<0.05; **, *P*<0.01).

It proved difficult to completely remove endogenous DAG molecular species from microsomes expressing *Ea*DAcT without adversely affecting enzyme activity. Further, changes in the solubility of DAG according to the nature of their acyl chains raised concerns about the accessibility of these substrates to the enzyme when added to an aqueous reaction mix. Therefore to determine the DAG preference of *Ea*DAcT, we performed selectivity assays instead of specificity assays. Indeed, as enzymes are typically exposed to multiple substrates *in vivo*, such selectivity assays might be more relevant to better understand the substrate preference of the enzyme. Monounsaturated DAG molecular species containing sixteen and eighteen carbon fatty acids were found to be the dominant species in yeast microsomes expressing *Ea*DAcT (Figure 2A). *In vitro* selectivity assays were performed for these DAG species by incubating with different concentrations of [1,2-^13^C] labelled acetyl-CoA. ESI-MS quantification of the nascent [1,2-^13^C] acetyl-TAG molecular species produced *in vitro* revealed that polyunsaturated DAG molecular species were preferentially acetylated over monounsaturated molecular species (Figure 2B). For example, the acetyl-TAG 36:2^1^ was more abundant than its monounsaturated counterpart 36:1, despite the fact that the DAG precursor for the latter (DAG 34:1) was available in larger quantities than that for the former (DAG 34:2; Figure 2A). Similar trends can be observed for the accumulation of acetyl-TAGs with 34 or 38 carbons. DAG species containing only saturated fatty acids were not incorporated at all in acetyl-TAGs (Figure 2B). This might be due to their very low abundances in microsomes (Figure 2A), the low preference of *Ea*DAcT for these DAG, or a combination of both effects. These results suggesting that *Ea*DAcT preferentially acetylates DAG species with higher unsaturation levels than those with lower saturation levels are consistent with the high incorporation of polyunsaturated fatty acids in the acetyl-TAGs naturally found in the endosperm of *E. alatus* [20] and transgenically produced in Camelina seeds expressing *Ea*DAcT [10].

### *Ea*DAcT can acetylate DAG containing a range of acyl chain lengths

**Figure 3:**
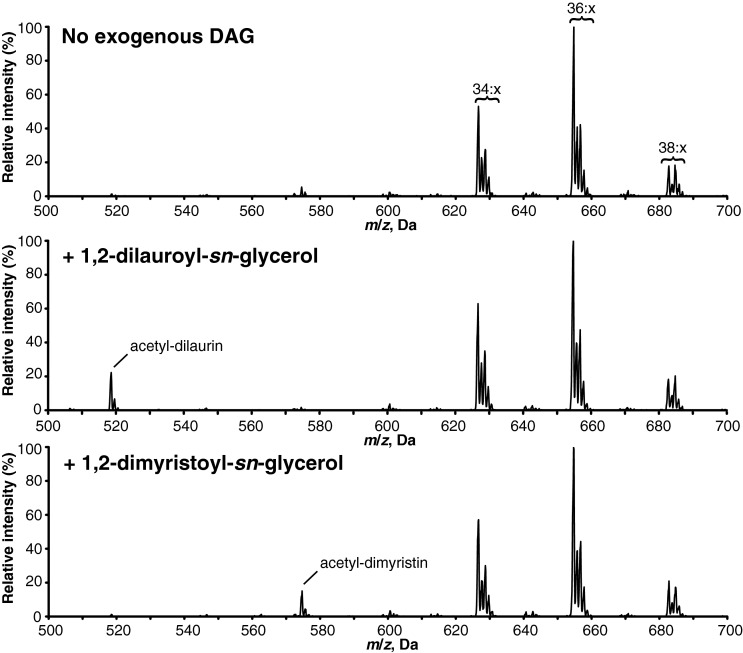
*Ea*DAcT can acetylate DAG containing different chain length fatty acids Positive ESI mass spectra obtained from the neutral loss of ammonium acetate from lipid products resulting from incubating microsomes containing *Ea*DAcT with acetyl-CoA and different exogenous DAG substrates. Peaks correspond to *m*/*z* values of the [M+NH_4_]^+^ adduct of the intact acetyl-TAG molecule. The number of acyl carbons in each series of acetyl-TAG molecules is indicated; for clarity, the number of double bonds (*x*) is not defined.

Previous work monitoring the accumulation of acetyl-TAGs in yeast expressing *Ea*DAcT revealed the presence of acetyl-TAGs molecular species containing medium chain fatty acids (MCFA; [13]). To confirm these results, MCFA-containing DAGs were added to *in vitro* acetyltransferase assays and the products analysed using ESI-MS. The resulting spectra showed peaks corresponding to the presence of 3-acetyl-1,2-dilauroyl-*sn*-glycerol and 3-acetyl-1,2-dimyristoyl-*sn*-glycerol when microsomal *Ea*DAcT was incubated with exogenous DAG containing lauric acid and myristic acid respectively (Figure 3). The other acetyl-TAG molecular species present in the spectra result from the acetylation of DAG present in the yeast microsomes. Under these conditions, the amount of acetyl-TAGs derived from exogenous DAG containing MCFA was always less than those derived from the endogenous DAG. These results might be caused by differential access of *Ea*DAcT to the exogenous DAG or by a low preference for these more saturated molecules.

### *Ea*DAcT is unable to acetylate other positions on the glycerol backbone of glycerolipids

**Figure 4:**
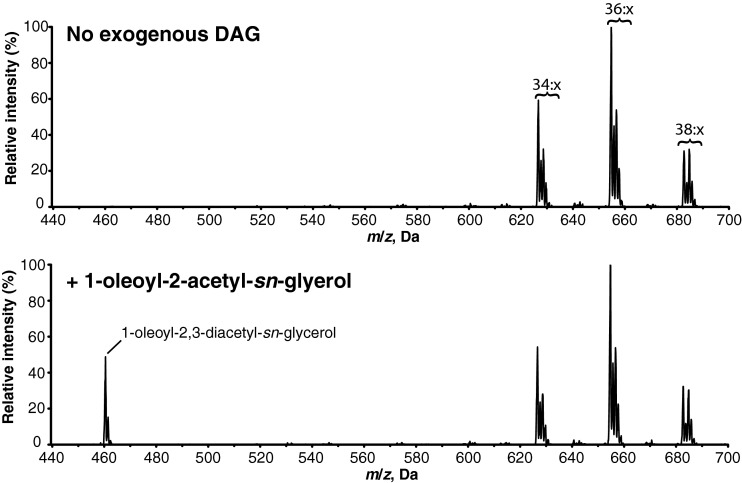
*Ea*DAcT can acetylate DAG containing an *sn*-2 acetyl-group Positive ESI mass spectra obtained from the neutral loss of ammonium acetate from lipid products resulting from incubating microsomes containing *Ea*DAcT with acetyl-CoA and 1-oleoyl-2-acetyl-*sn*-glycerol. Peaks correspond to *m*/*z* values of the [M+NH_4_]^+^ adduct of the intact acetyl-TAG molecule. The number of acyl carbons in each series of acetyl-TAG molecules is indicated; for clarity, the number of double bonds (*x*) is not defined.

Optical rotation studies and NMR analysis demonstrated that both naturally and transgenically produced acetyl-TAGs possess an *sn*-3 acetate group [1,3], consistent with the ability of *Ea*DAcT to acetylate the *sn*-3 hydroxyl group of *sn*-1,2-DAG (Figure 3; [1]). To determine whether *Ea*DAcT is also capable of acetylating the *sn*-2 position on a glycerolipid backbone, microsomes containing the enzyme were incubated with *sn*-1,3-DAG. However, no acetylated reaction products were detected (results not shown). We were also interested to know whether *Ea*DAcT could acetylate the available hydroxyl groups on the backbone of monoacylglycerol (MAG). Similar to the result with *sn-*1,3 DAG, no acetylated products were detected when *Ea*DAcT microsomes were incubated with either *sn*-1 MAG or *sn*-2 MAG (results not shown). These results distinguish *Ea*DAcT from DGAT1 which was shown to be able to acylate these types of lipids *in vitro* [21]. *Ea*DAcT was capable of acetylating 1-oleoyl-2-acetyl-diacylglycerol to form a diacetylated TAG product (Figure 4). As 1-oleoyl-2-acetyl-sn-glycerol is structurally similar to *sn*-1-MAG in that the *sn*-2 position does not contain a long acyl group, this result suggests that the length of *sn*-2 acyl chain is unimportant relative to the requirement that the *sn*-2 hydroxyl group be acylated. Further, as *sn*-2-MAG are also not substrates for *Ea*DAcT, the *sn*-1 hydroxyl group also needs to be occupied in order for the *sn*-3 position to be acetylated by the enzyme. Thus *Ea*DAcT is only capable of acetylating the *sn*-3 position of glycerolipids where the other hydroxyl groups on the backbone are already esterified to other acyl groups.

### *Ea*DAcT can acetylate aliphatic chain fatty alcohols *in vitro*

**Figure 5:**
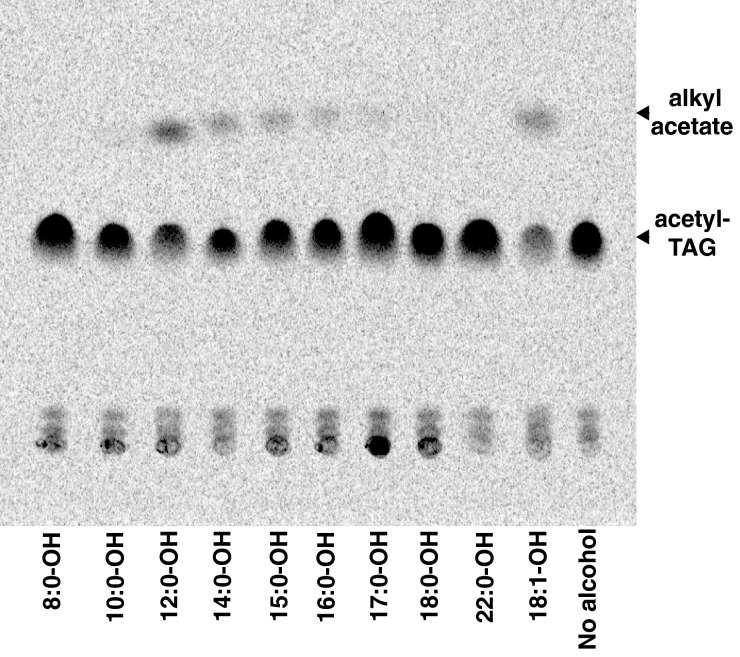
*Ea*DAcT can acetylate a range of fatty alcohols *in vitro* TLC separation of total lipids extracted yeast microsomes expressing *Ea*DAcT incubated with [1-^14^C] acetyl-CoA and 125 μM of different chain length alcohols. Fatty alcohols are denoted using *x*:*y* where *x* indicates the number of carbons and *y* the number of double bonds.

Based on protein sequence, *Ea*DAcT clusters with the jojoba wax synthase and the Arabidopsis sterol acyltransferase [1,10]. Hence, the activity of *Ea*DAcT towards various alcohols and sterols was tested under various conditions. In multiple *in vitro* assays, we did not detect any radiolabelled sterol acetates derived from endogenous sterols in microsomes (Figure 5, Supplementary Figures S1 and S2) [1]. In addition, we also added cholesterol to *in vitro* acetylation reactions, but were unable to detect the formation of cholesterol acetate (results not shown). However, consistent with previous work [11,12], when incubated with different fatty alcohols, *Ea*DAcT was capable of acetylating these compounds to form alkyl acetates. The addition of DMSO was found to be necessary to provide the activity due to the insoluble nature of fatty alcohols in an aqueous buffer system (Supplementary Figure S2A). The alkyl acetate product was much less abundant than acetyl-TAGs synthesized from the acetylation of endogenous DAG. This might either be due to lower reactivity of *Ea*DAcT towards alcohols than towards DAG, or low solubility and accessibility of alcohol substrate to microsomal *Ea*DAcT. To address the latter, a number of detergents at concentrations which did not result in loss of enzyme activity were tested to increase the formation of alkyl acetates (Supplementary Figure S2B). Maximal product accumulation was still achieved when DMSO was used, followed by the use of non-ionic detergents in the order brij-35 (0.3%) > NP-40 (0.005%) > OTG (0.05%) > OBG (0.025%). Addition of the ionic detergent sodium deoxycholate was not effective in producing alkyl acetate product (Supplementary Figure S2B). *Ea*DAcT produced the most alkyl acetate between a pH of 7.0–8.5 with maximum oleyl acetate synthesis at a pH 7.5 (Supplementary Figure S2C). As this pH is also optimal for the formation of acetyl-TAGs (Supplementary Figure S1B), we speculate that both activities probably use the same group of amino acids in their catalytic mechanism. Formation of oleyl acetate increased sharply with the oleyl alcohol substrate concentration from 25 to 125 μM. However, a gradual decrease in activity was observed when the oleyl alcohol concentration was increased from 125 to 1000 μM probably due to detergent effects of the hydrophobic fatty alcohol substrate (Supplementary Figure S2D).

Once the assay conditions were optimized, the effect of chain length on product formation was investigated by incubating *Ea*DAcT microsomes with [1-^14^C] acetyl-CoA and different alcohols with carbon chain lengths varying from 8 to 22. Similar to recent observations [12], the highest amounts of alkyl acetates were produced with dodecyl alcohol, with activity decreasing as the length of the acyl chain increased, such that no product was detected with stearyl and behenyl alcohols (Figure 5). Further, no products were detected for saturated alcohols with a chain length less than ten carbons, suggesting a minimum size of 12 carbons for activity. Oleyl alcohol was acetylated to a greater extent than its saturated counterpart. This preference for more unsaturated alcohols is again consistent with previous work [12]. Aromatic alcohols such as cinnamyl alcohol, benzyl alcohol and 3-phenyl-1-propanol were also tested as substrates but no acetylated products were detected (results not shown). The ability of *Ea*DAcT to acetylate fatty alcohols is not too surprising given the phylogenetic similarity of the enzyme to the wax synthase responsible for the synthesis of wax esters in jojoba seeds [1,10]. Further, other MBOATs have also demonstrated promiscuity with regard to acyl acceptor specificity. In particular, it is interesting to note that like *Ea*DAcT, the human DGAT1 enzyme has been shown to acylate fatty alcohols in addition to DAG [21]. It is therefore tempting to speculate that at least for MBOATs, active sites capable of recognizing DAG can also accommodate fatty alcohols, though more detailed structural information is needed to confirm this idea.

### *Ea*DAcT can synthesize alkyl acetates *in vivo*

**Figure 6:**
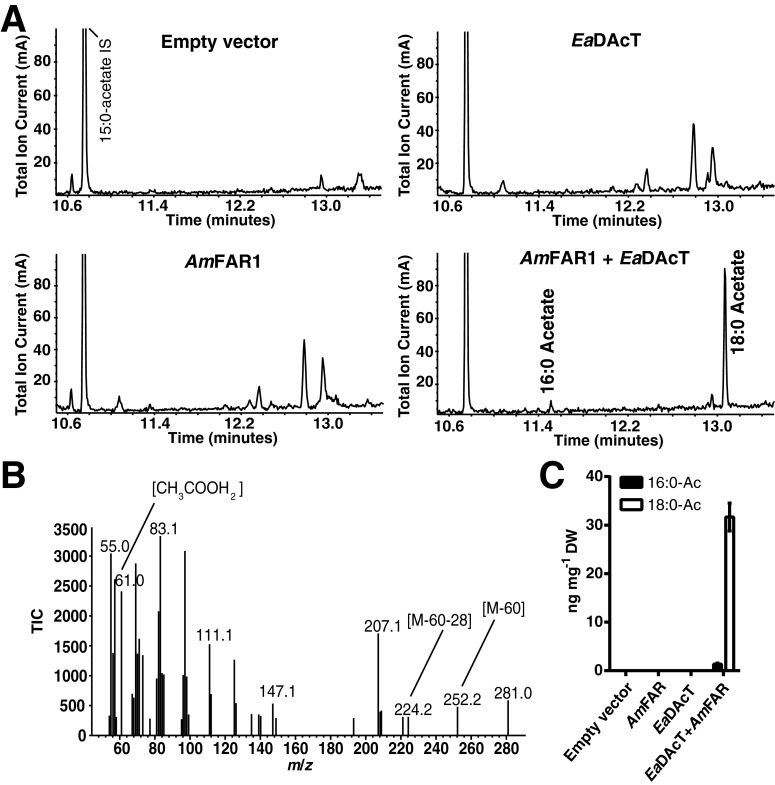
*Ea*DAcT can acetylate fatty alcohols *in vivo* (**A**) GC–MS chromatograms of alkyl acetates purified from H1246 yeast expressing the empty vector pESC-URA or combinations of *Am*FAR1 and *Ea*DAcT. (**B**) Electron impact mass spectrum of the stearyl acetate peak (retention time=13.1 min) labelled with diagnostic fragment ions. (**C**) Quantification of alkyl acetates from (**A**). Values represent mean ± S.D. for three biological replicates.

The ability of *Ea*DAcT to synthesize alkyl acetates *in vivo* was also examined by heterologous coexpression of *Ea*DAcT and the honeybee fatty acyl-CoA reductase *Am*FAR1, which was previously reported to produce fatty alcohols when expressed in yeast [16]. GC–MS analysis of lipids from yeast expressing both *Ea*DAcT and *Am*FAR1 revealed two novel peaks in the chromatogram that were not present in the empty vector control or when the genes were expressed individually (Figure 6A). The electron impact mass spectra of both peaks matched the characteristic fragmentation patterns of palmityl acetate and stearyl acetate (Figure 6B). No other alkyl acetate products were detected, consistent with observations that *Am*FAR1 reduces only saturated fatty acids to alcohols [16] and thus only palmityl and stearyl alcohols were detected in lipids extracted from yeast expressing *Am*FAR1 (Supplementary Figure S3A). Relatively low amounts of alkyl acetates were produced by yeast expressing *Am*FAR1 and *Ea*DAcT (Figure 6C) compared with the amounts of fatty alcohols potentially available (Supplementary Figure S3B). This result might indicate the low preference of *Ea*DAcT for these long-chain saturated alcohols, in agreement with the *in vitro* assay results (Figure 5). Likewise, previous attempts to produce alkyl acetates *in vivo* using a transient expression system in tobacco leaves also resulted in a low conversion rate, particularly for saturated fatty alcohols [12].

In conclusion, these results confirm that *Ea*DAcT preferentially utilizes acetyl-CoA to acetylate *sn*-1,2 DAGs but that other acyl-donor and acyl-acceptor substrates can be used with low efficiency. Our work suggests that it should be possible to synthesize acetyl-TAG with a range of fatty acids at the *sn*-1 and -2 positions, thus further altering the physical properties of these unusual TAG molecules. For example, because viscosity appears to primarily depend on molecular weight [22], it might be possible to synthesize acetyl-TAG with further reductions in viscosity through the acetylation of DAG containing MCFA. Here we show that *Ea*DAcT is capable of acetylating such DAG molecules, albeit with lower efficiency. The acyl acceptor preference of the enzyme therefore suggests that it might be worthwhile considering the incorporation of unsaturated MCFA into acetyl-TAG instead. Additionally, because *Ea*DAcT is incapable of using acyl-CoA molecules longer than six carbons, it will not use MCFA acyl-CoA to acylate DAG when expressed in a background capable of producing such molecules. This better understanding of the substrate specificity of *Ea*DAcT will therefore be helpful in efforts to rationally engineer the synthesis of desired target molecules in transgenic plants.

## Footnotes

^1^ For lipid molecular species, the number of acyl carbons (*x*) and double bonds (*y*) is indicated by *x* : *y*.

## Abbreviations

acetyl-TAG: 3-acetyl-1,2-diacylglycerol
DAG: diacylglycerol
DGAT: diacylglycerol acyltransferase
*Ea*DAcT: *Euonymus alatus* diacylglycerol acetyltransferase
FAME: fatty acid methyl esters
FAR: fatty acyl-CoA reductase
GOAT: ghrelin *O*-acyltransferase
MAG: monoacylglycerol
MBOAT: membrane-bound *O*-acyltransferase
MCFA: medium chain fatty acid
TAG: triacylglycerol
TLC: thin layer chromatography
